# Evolutionary Links Between Skull Shape and Body Size Suggest Allometric Forces and Selection at Work in a Generalist Group of Lizards

**DOI:** 10.1002/ece3.70594

**Published:** 2024-11-17

**Authors:** Julio A. Rivera, Jesualdo A. Fuentes‐G., Emília P. Martins

**Affiliations:** ^1^ School of Life Sciences Arizona State University Tempe Arizona USA; ^2^ Department of Biological Sciences Florida International University Miami Florida USA

**Keywords:** body size, CT scans, *Sceloporus*, skull shape

## Abstract

The vertebrate skull is a complex structure, and studies of skull shape have yielded considerable insight into the evolutionary forces shaping specialized phenotypes in organisms as diverse as bats, frogs, and fossorial animals. Here, we used phylogenetic comparative analyses of CT scans of male skulls from 57 species of *Sceloporus* lizards to explore patterns of skull evolution in a group of generalist taxa. We found that most interspecific variation is in terms of skull elongation such that some species have long, narrow skulls, whereas others exhibit more compact and robust skulls. We also found strong links to overall body size, with evolutionary shifts to larger bodies being associated with more compact skulls and slower evolutionary rates. This is the opposite of the pattern in most mammals in which larger bodied species have longer snouts, and more like the pattern in frogs in which function has played a more important evolutionary role. Also, unlike other vertebrates, the jaw, anterior, and posterior parts of the *Sceloporus* skull are largely integrated, having evolved independently of each other only to a limited, albeit significant, degree. Our results emphasize the importance of body size in the evolutionary shaping of the skull and suggest that additional studies of behavioral function in a generalist group are warranted.

## Graphical abstract

We use geometric morphometrics and phylogenetic comparative methods to study skull shape evolution in *Sceloporus* lizards. We found that overall body size is driving shape evolution; however, sexual selection seems to play a role. Moreover, the skull has evolved as a single unit with little modularity occurring.

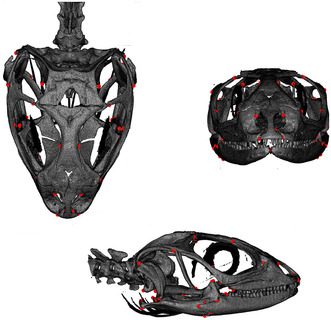

## 1 Introduction

The size and shape of the vertebrate skull hold a great deal of information about the complex forces that have acted on it simultaneously and sequentially through evolutionary time. Skulls sometimes evolve as integrated units, and at others as separately evolving modules. At times, these forces may allow species to explore new morphospaces and novel phenotypes, as is the case with the snouts of some anole species where the cranial modules are labile, rather than conserved, and reflect functional demands like the convergence of snout elongation found in the *carolinensis* and *hendersoni* series (Sanger et al. 2012). Developmental processes (e.g., heterochrony—a change in the rate of developmental processes relative to the ancestral state) and body size can also constrain the evolution of skull shape, such that larger mammals (Cardini and Polly 2013) and pythons (Esquerré, Sherratt, and Keogh 2017) have longer faces than do smaller mammals. Strong functional constraints can sometimes reverse this process in specialized animals, as in the paedomorphic skulls of lizards from arid environments (Hipsley and Müller 2017), fossorial lizards (Barros, Herrel, and Kohlsdorf 2011), snakes (Da Silva et al. 2018; Watanabe et al. 2019), and strong‐jawed frogs (Carla Bardua et al. 2021; Isip, Jones, and Cooper 2022).

Modules are groups of traits that can change independently during development. Examples of this include the face and the neurocranium of domestic dogs (Drake and Klingenberg 2010) and other mammals (Marroig et al. 2009; Shirai and Marroig 2010). Yet, in other vertebrates, the mandible often evolves as a separate module, as in bats (Arbour, Curtis, and Santana 2019) and felids (Christiansen 2008). Modularity can often drive phenotypic change facilitating the exploration of novel morphospace, and can promote rapid diversification, as in the cranial morphology of some *Anolis* lizards (Sanger et al. 2012), caecilians (Bardua et al. 2019), and frogs (Bardua et al. 2020). Alternatively, the integration of traits can also promote morphological disparity possibly by coordinating the response of said traits within a unit to a selective pressure leading to the exploration of novel morphologies (Klingenberg 2010). For example, both placental and marsupial mammals show patterns of integration and higher levels of disparity compared to what would be expected under a stochastic process (Goswami et al. 2014).

Body size fundamentally impacts many aspects of an organism's biology (Bergmann 1847) with allometric relationships often explaining the evolution of morphology, ecology, lifespan, and even reproductive output (Calder 2001). In fact, evolutionary shifts in body size are often associated with substantial changes in shape (Gould 1966), which can be predictable because of developmental integration (Klingenberg 1998). For example, in raptors, skull and beak shape are integrated and regulated significantly by body size and developmental constraints so that body size accounts for nearly 80% of beak shape variation (Bright et al. 2016). In mammals (Cardini and Polly 2013) and some lizards (Gray et al. 2019; Hipsley and Müller 2017; Urošević, Ljubisavljević, and Ivanović 2013), body size may constrain changes in facial morphology and the brain case during development so that larger animals have longer faces while smaller ones have shorter faces. In other taxa, evolutionary changes in body size and development do not constrain, and may instead enhance phenotypic evolution, as in Indo‐Pacific shore fishes for which changes in body size explain < 3% of interspecific variation in body shape (Friedman et al. 2019). In fire salamanders, body size and heterochrony do not explain rapid shifts in jaw morphology (Alarcón‐Ríos et al. 2020), and in *Liolaemus* lizards, body shape has been much more labile than body size over evolutionary time frames (Edwards et al. 2022).

The lizard genus *Sceloporus* presents an ideal opportunity to investigate the patterns of variation across diverse taxa and the roles modularity and body size have played in generating this variation. *Sceloporus* lizards are endemic to North America, representing over 90 species (Leaché et al. 2016) that use a variety of habitats from beaches to high‐elevation grasslands and forests (IUCN 2018). As the hallmark iguanian lizard, the genus has been well studied from behavioral (Carpenter 1978; Hews and Martins 2013), ecological (Sinervo et al. 2010), biogeographical (Lawing et al. 2016; Rivera, Lawing, and Martins 2020), genomic and phylogenetic perspectives (Leaché et al. 2016; Wiens et al. 2010) allowing for detailed comparative studies. These lizards vary tremendously in size from ~40 to ~120 mm snout‐to‐vent length (SVL) (Rivera et al. 2021), and interspecific differences are the result of a complex mix of intrinsic differences in life history and genetics, as well as multiple external selective forces (Jiménez‐Arcos, Sanabria‐Urbán, and Cueva del Castillo 2017; Leaché et al. 2016; Lopez‐Alcaide, Cuateta‐Bonilla, and Macip‐Ríos 2020).

Here, we used geometric morphometrics and phylogenetic comparative analyses to understand the forces, allometric patterns, and selection, driving shape evolution on *Sceloporus* lizard skulls. First, we study the statistical and evolutionary impact of allometry on the shape of the skull and ask whether the skull and body size are evolutionarily decoupled. Second, we identify the primary axes of variation of the skull and ask whether these axes remain intact when we incorporate evolutionary history. Last, we investigate whether shape variation is explained by modularity, explicitly testing three competing module schemes, or phenotypic integration where the skull is evolving as a single unit.

## 2 Methods

### 2.1 Specimens, Scanning, and Digitization

We borrowed *Sceloporus* specimens from five collections: the Museum of Vertebrate Zoology at the University of California Berkeley, the Amphibian and Reptile Diversity Research Center at the University of Texas at Arlington, the Museum of Southwestern Biology at the University of New Mexico, the Museum of Natural History at the University of Colorado Boulder, and the Burke Museum at the University of Washington. We then used microtomography (μCT) scanning to visualize and measure the cranial morphology of one male specimen from each of 57 species of *Sceloporus* lizards (Table S1). We decided to choose only males as some *Sceloporus* species show sexual size and shape dimorphism. Although this approach allows us to reduce the intraspecific variability, it also ignores real biological differences among the sexes that can provide additional insight into evolutionary processes. We chose the largest available male specimen for each species and compared overall body size with measures from the literature for each species to ensure that we were comparing adults. For *Sceloporus edwardtaylori* , however, we had access only to a sub‐adult male specimen.

Prior to scanning, we tagged specimens with unique radio‐opaque labels, wrapping lizards of roughly the same size in 70% ethanol‐soaked cheesecloth and packing them tightly into a PLA (polylactic acid)‐plastic cylinder (see Buser et al. (2020) for details). We then scanned the specimens using the Bruker Skyscan 1173 at the Karel F. Liem Bio‐Imaging Center at Friday Harbor Laboratories. We set the μCT scanner at 65 kV and 123 μA with a voxel size ranging from 17.1 to 33.5 μm. We focused on dense bone tissue, and so did not stain the specimens with additional chemical agents. We converted the reconstructed scans to the dicom (.dcm) file format and used Real3D Scanner (Ullah 2019) software to render and convert the 3D images to .ply file format.

For analyses that consider the evolution of skull shape in a phylogenetic context, we pruned the Leaché et al. (2016) time‐calibrated phylogeny to match the taxa for which we had 3‐dimensional data. Due to recent changes in taxonomic nomenclature, this phylogeny does not include four of our target species ( *Sceloporus cozumelae* , *S. hartwegi*, *S. prezygus*, and *S. utiformis* ). *S. hartwegi*, *S. prezygus*, and *S. utiformis* were recently elevated from subspecies to species, so we considered them sister taxa to their closest relatives: *S. taeniocnemis* , *S. serrifer* , and *S. grandaevus* , respectively. We considered *S. cozumelae* sister taxon to *S. variabilis* , following the Wiens et al. (2010) phylogeny. Although there is considerable debate regarding the phylogenetic history of *Sceloporus*, there is consensus regarding the primary subclades, and the most recent phylogeny (Leaché et al. 2016) offers clear insight into the chromosomal and genomic mechanisms that underlie previous misunderstandings. All our comparative analyses assume that this single phylogeny represents the evolutionary relationships among species.

### 2.2 Body Size as a Predictor of Shape

**FIGURE 1:**
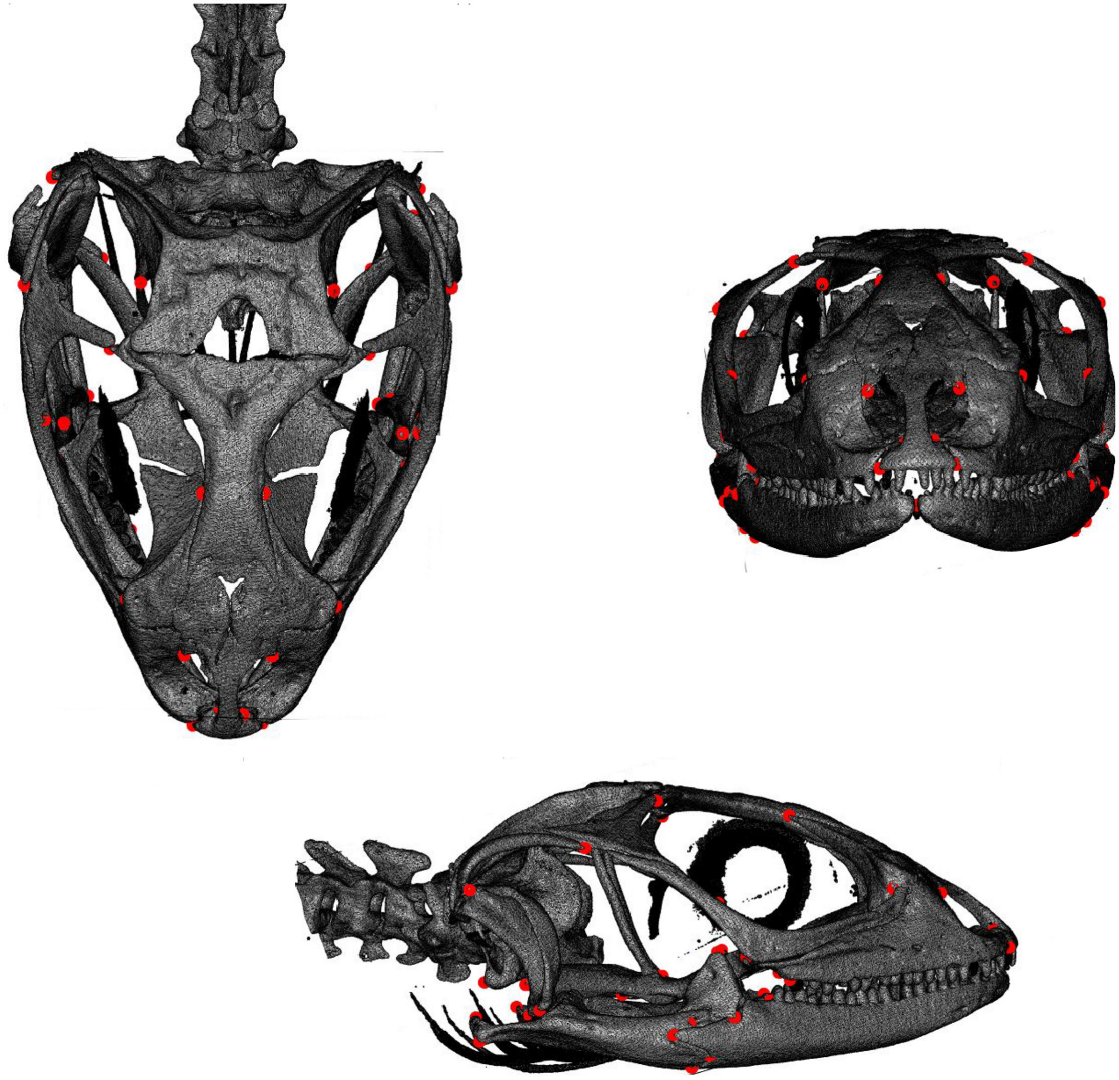
Illustration of the 69 landmarks (red points) that we used to digitize the shape of male *Sceloporus* skulls.

We scored 69 landmarks across each skull (Figure 1, Table S2) using the package “geomorph” (Adams et al. 2017) in R (R Core Team 2017), and choosing landmarks representative of the overall shape of the skull and that were readily identifiable in all species. In addition, we derived linear measures from the 3‐dimensional data by measuring the distance between landmarks that represent total skull length, width, and height (see Table S2 for details). We also measured body size as snout‐to‐vent length, or SVL, from the preserved specimens. In many of the analyses (identified below), we divided the linear measures by body size to obtain relative skull dimensions. For some analyses identified below, we designated a species as “small” when the mean SVL of the species was < 56 mm, “medium” if 57–74 mm, and “large” if > 75 mm (Rivera et al. 2021).

To describe interspecific variation in skull shape, we began with a Generalized Procrustes Analysis (geomorph: gpagen). The Procrustes Analysis places each specimen at the origin, scales it to unit‐centroid size, and rotates until the coordinates of corresponding points on other specimens align as closely as possible (Gower 1975; Rohlf and Slice 1990). Specifically, we predicted that body size would have a large impact on skull shape so that species similar in size have similarly shaped skulls.

Additionally, we used several comparative approaches to test whether evolutionary changes in body size have guided skull evolution. First, we used linear regression accounting for evolutionary history to determine how well centroid size predicted skull shape. We also performed a Procrustes ANOVA accounting for phylogenetic relationships (geomorph: ProcD.pgls) and performed a pair‐wise comparison between the body size classes. In addition, we used Hansen (1997) adaptation‐inertia model to estimate the relative importance of body size in shaping skull evolution in a complex selective regime. The adaptation‐inertia model assumes that phenotypes are subject to multiple selective pressures and estimates the importance of a single feature in shaping interspecific variation. To do so, we extracted time spent evolving in a particular body size category (small, medium, or large) from the ancestral reconstruction in Rivera et al. (2021), and used this as a predictor variable (*X*) in separate regression models predicting the evolution of skull dimensions (*Y* = relative skull length, width, and height). For each response measure, we then compared the fit of three models: neutral evolution (Brownian motion), single regime (global optimum), and three regimes (small, medium, and large body sizes). Model parameters were estimated using SLOUCH (Kopperud et al. 2019), and we compared model fits using AIC values. These univariate analyses offer unique insight into the long‐term impacts of body size on skull evolution. Because we predicted that body size would have a large impact on skull evolution, we expect that each body size category will have a different optimum for skull length, width, and height so that the three‐regime model will best fit our data.

We also considered the impact of body size shifts on the rate of skull evolution, testing whether major evolutionary changes in body size were associated with increases in the diversification of lizard skulls. We inferred evolutionary skull shape optima and rates of skull shape evolution among size classes using AIC_c_ to compare models of phenotypic change. We also inferred the evolutionary rates of the linear measures (ratios of skull length, width, and height) for the three size categories, using the maximum likelihood model fit by the R version of “Brownie” (O'Meara et al. 2006), “brownie. lite” implemented in “phytools” (Revell 2012). We compared whether traits were better modeled under a single rate or multiple rates of evolution and used log‐likelihoods and a likelihood ratio test to evaluate our results.

### 2.3 Identifying Primary Axes of Variation

To identify the axes of variation, we performed three types of principal components analyses (PCA) on the new set of coordinates: (1) a traditional non‐phylogenetic PCA, (2) a PCA that accounts for phylogenetic non‐independence (Phy‐PCA: Revell 2009), and (3) a PCA that emphasizes phylogenetic signal by aligning interspecific data with phylogenetic signal (PACA: Collyer and Adams 2021). Here, we aim to address whether evolutionary history and phylogenetic signals have played a role in skull shape evolution. We chose to perform the Phy‐PCA (geomoprh: gm.prcomp) because data reduction procedures often do not account for nonindependence among species. The Phy‐PCA takes into account the phylogeny to statistically correct for the nonindependence in data reduction procedures, like a PCA. Additionally, we performed a PACA (geomoprh: gm.prcomp) which aligns the shape data to the phylogenetic signal. The difference between the Phy‐PCA and PACA is that the Phy‐PCA aligns the principal eigenvectors independently of the phylogenetic signal while a PACA maximizes variation in a direction that describes the phylogenetic signal and leaves intact the Euclidean distances between species in the morphospace.

Finally, we estimated the degree to which skull shape has evolved in concert with phylogeny by estimating the degree of phylogenetic signal (geomorph: physignal). We estimated the multivariate version of the *K*‐statistic, *K*_mult_ (Adams 2004; Blomberg, Garland, and Ives 2003) based on the Procrustes analysis of skull shape (Adams et al. 2017). We also used Pagel's *λ* (Pagel 1999) to estimate the degree of phylogenetic signal (phytools: phylosig) in linear skull dimensions (Revell 2012). We chose to use the *K*_mult_ statistic for the shape data because it can infer the phylogenetic signal of high‐dimensional traits. Pagel's *λ* was used for the linear measurements as they are 2‐dimensional. The authors note that these two statistics are not interchangeable as the *K* statistic is akin to the proportion of the covariance that is due to phylogenetic history while *λ* is akin to scaling the branch lengths on a phylogeny under a Brownian motion model. However, both approaches measure the impact of phylogeny on trait evolution. In both cases, we used randomization tests to compare estimates of phylogenetic signal to a value of 1.0 (expected under a Brownian Motion model).

### 2.4 Modularity and Integration of the Skull

Last, we tested whether the *Sceloporus* skulls were evolving as a single unit (geomorph: integration. test) or as multiple modules (geomorph: modularity. test) by comparing the fit of four models. First, we followed and Sanger et al. (2012) to fit two models based on cellular processes acting during skull development: an “*Anolis*” model that differentiates between anterior (snout) and posterior (brain case) regions of the skull, and a “mammal” model which divides the skull into anterior and posterior sections but includes more of the central skull in the anterior (“facial”) module. Third, we fit a tripartite model, isolating the mandible as a separate unit from the anterior and the posterior part of the skull, as we might expect if bite force (i.e., linked to diet or male–male fighting) has shaped skull evolution (Table S2). Last, we fit a null model of no modules. Here, we predicted that the *Sceloporus* skull would fit the tripartite model as jaw shape is likely to be influenced by diet, the anterior skull by behavior and communicative signaling, and the posterior skull by habitat use. For each model, we estimated covariance ratios (CR) and effect sizes (*Z*_CR_) to infer the extent to which the sections have evolved as independent modules or in concert. Low covariance ratios (CR = 0) indicate high modularity, with tight associations within each module, but low correlations across modules. High covariance ratios (CR = 1) indicate less modularity, either because there are tight links between modules or because connections within modules are weak. We estimated covariance ratios (CR) and compared each to a neutral distribution of possible *CR*s using permutation tests as implemented in the package “geomorph” (Adams 2004; Adams and Collyer 2019). Then, we tested whether the modules are evolving as independent groups or integrated, evolving in concert. We did this both by quantifying module integration between the groups and then quantifying phylogenetic morphological integration under Brownian motion using “geomorph.” The integration test also calculates partial least squares (PLS) between the designated modules, which we report along with the alpha.

## 3 Results

### 3.1 Body Size as a Predictor of Shape

**FIGURE 2:**
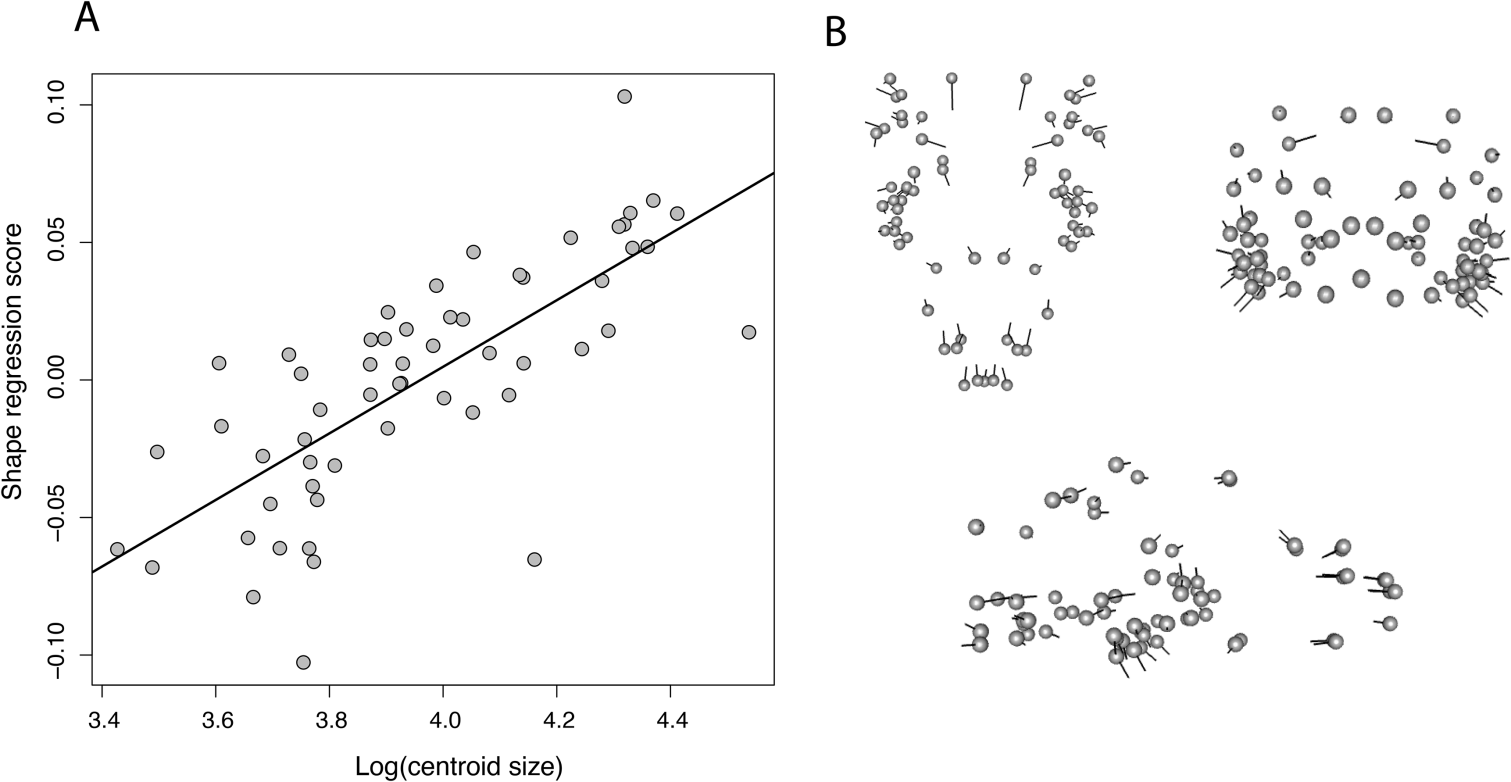
(A) A regression of log‐transformed centroid size against the regression scores fitted with a trend line and (B) depicts the displacement in shape between small *Sceloporus* species (points) and large species (the vectors).

Centroid size was a good predictor of skull shape (Figure 2A; *F* = 9.2, df = 1, *R* ^2^ = 0.15, *p* < 0.01). We found that allometric patterns of shape indicated that larger bodied species have shortened anterior and posterior skulls, wider posterior skulls, and taller skulls compared to small‐bodied species (Figure 2B). Additionally, body size was a significant predictor of overall skull shape in the phylogenetic Procrustes ANOVAs (body size categories: *F* = 4.8, df = 2, 55, *p* = 0.02; skull centroid size: *F* = 8.8, df = 1, 55, *p* = 0.01) so that larger and medium species differed (*p* < 0.01), large and small species differed (*p* < 0.01), but medium and small species only marginally differed (*p* = 0.09) from each other. As shown also by the adaptation‐inertia models (Table 1), evolutionary time spent as small‐, medium‐, or large‐bodied species explained a substantial amount of the variation in skull dimensions, including 12% of relative skull length and 9% of skull width. In terms of linear optima, small species had a relatively longer skull length optimum while large species had a relatively shorter skull length optimum. We found no differences in the model with a single optimum (global optima) versus that with multiple optima for skull width. However, the multi‐optima model showed that smaller bodied species had relatively narrow skulls while larger bodied species had relatively wider skulls. Lastly, we found that skull height was evolving in response to a single global optimum rather than a multi‐optima model or neutral evolution.

**TABLE 1:** Parameter estimates and one standard error (in parentheses) from Adaptation‐Inertia models estimating the degree to which time spent evolving in each size category (small, medium, or large body size) predicts the evolution of male skull dimensions. Three competing hypotheses were tested: Brownian motion, a single regime (no difference between body size categories: OU1), and three regimes (different optimal values for small‐, medium‐, and large‐bodied contexts: OU size). BM models never offered the best fit, so only ΔAIC values are reported. Values in bold indicate the best‐fit model.

	ΔAIC	Θ_small_	Θ_medium_	Θ_large_	Θ_global_	*r* ^2^
Relative skull length (BM ΔAIC = 65)						
OU1	34				0.24 (0.004)	2.6%
**OU size**	**0**	**0.25 (0.004)**	**0.24 (0.004)**	**0.23 (0.004)**		**11.7%**
Relative skull width (BM ΔAIC = 25)						
**OU1**	**0**				**0.18 (0.003)**	**13.4%**
**OU size**	**0**	**0.17 (0.004)**	**0.18 (0.004)**	**0.19 (0.004)**		**8.6%**
Relative skull height (BM ΔAIC = 24)						
**OU1**	**0**				**0.12 (0.002)**	**11.4%**
OU size	1	0.11 (0.003)	0.11 (0.002)	0.12 (0.002)		3.2%

In terms of differences in evolutionary rate, we found evidence that skull length has evolved more quickly in smaller bodied *Sceloporus* species than in medium‐ or large‐bodied species (Table 2). There were no significant differences in evolutionary rate estimates between lizard species in different size classes for any of the other skull measures. Similarly, the evolution of skull width and skull height was best explained by a single rate model (top of Table 2).

**TABLE 2:** Evolutionary rate estimates of the linear skull measures and one standard error (in parentheses). Likelihood scores for both single‐ and multi‐rate models are also shown. *p*‐values were calculated based on a *χ* ^2^ of the single‐ and multi‐rate models. Values in bold indicate the best model.

	Single‐rate model		Multi‐rate model				*p*
	*σ* ^2^	Likelihood	*σ* ^2^_Small body size_	*σ* ^2^_Medium body size_	*σ* ^2^_Large body size_	Likelihood	
Relative skull length	2.3 (0.44)	−176.2	**4.8 (1.7)**	**0.27 (0.11)**	**2.3 (0.82)**	**−164.9**	**< 0.01**
Relative skull width	**1.1 (0.19)**	**−145.1**	1.1 (0.89)	0.2 (0.06)	2.1 (1.36)	−152.7	**< 0.01**
Relative skull height	**0.5 (0.09)**	**−122.4**	0.7 (0.32)	0.07 (0.029)	0.7 (0.30)	−130.3	**< 0.01**

### 3.2 Primary Axes of Variation

**FIGURE 3:**
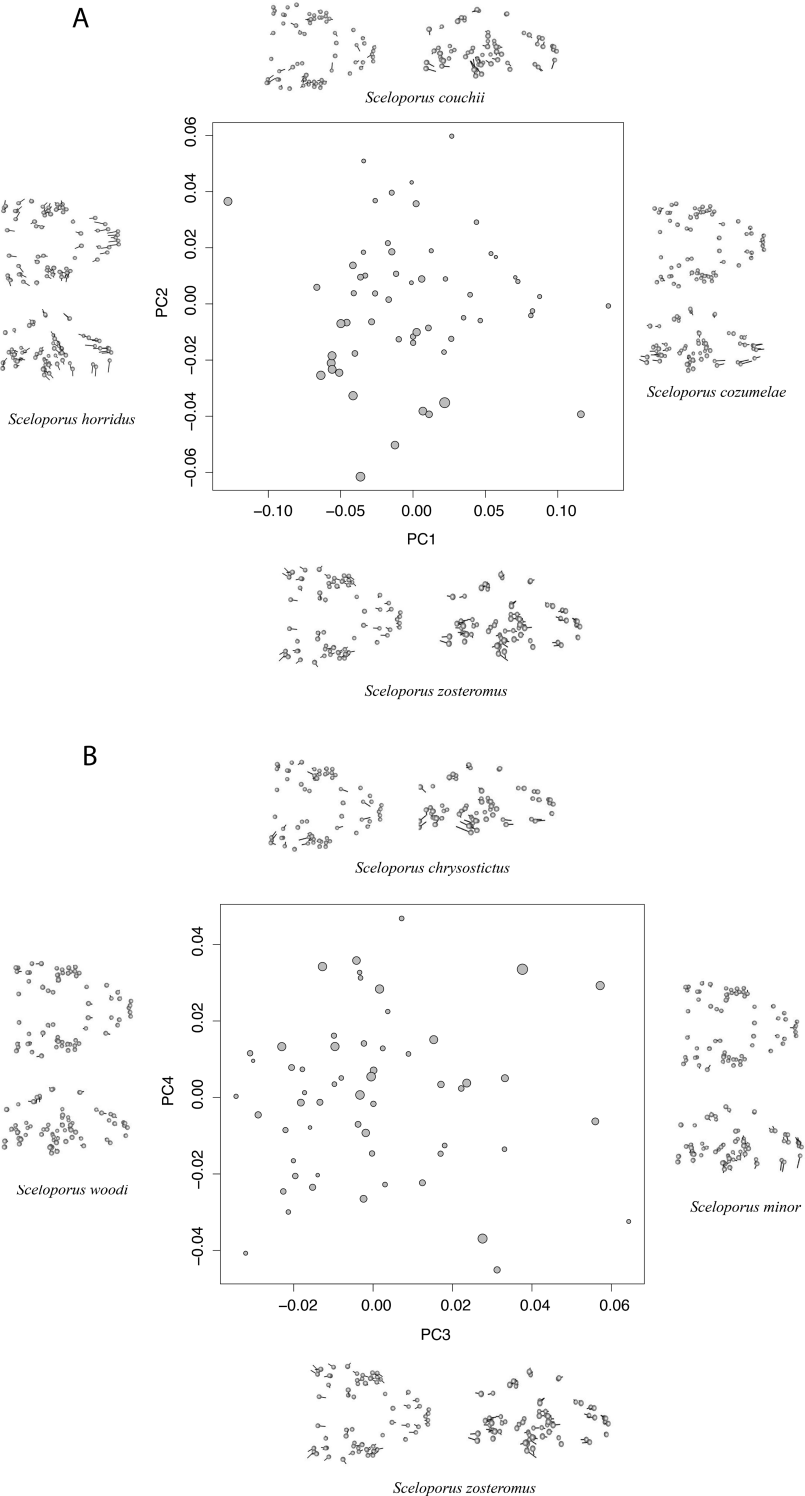
Results of Principal Components Analysis for male skull shape for measures of 57 *Sceloporus* lizard species. Panel (A) shows PC1 (snout length and jaw width) and PC2 (shortness of jaw length) while panel (B) illustrates interspecific differences in PC3 (snout height) and PC4 (shortness of center skull height). The size of the points is proportionate to the log‐transformed centroid size of the skulls.

Traditional PCA: *Sceloporus* skulls differed primarily across species in terms of their length and width. In a traditional, non‐phylogenetic PCA, most of the variation (34%) was described by the first PC axis, which was focused on the length of the skull, with positive loadings indicating longer skulls, and the width of the skull with positive loadings indicating a narrowing of the posterior skull and posterior jaw (Figure 3A). The second axis, PC2, representing 9% of the variation, primarily reflected differences in skull height and again, skull length. Positive PC loadings indicate taller anterior parts of the skull (snout) while the posterior skull was flatter. Additionally, species in this PC space also had overall shorter skulls in length and narrowing of the posterior skull (Figure 3A). The third axis, PC3, represented 7% of the variation, with positive loadings indicating taller anterior parts of the skull (snout) and a shortening in the overall skull length (Figure 3B). The fourth axis, PC4, represented only 6% of the variation, with positive values indicating a skull that was shorter in length and a narrowing of the posterior skull (Figure 3B). Additional axes represented only very small amounts of the interspecific variation in skull shape.

PACA: Incorporating the phylogeny in our PCA gave us nearly identical results as the traditional PCA but placed more variation into fewer axes. For example, the PACA that emphasized phylogenetic signal (Collyer and Adams 2021) put 42% of trait variation into the first PC axis, where positive values represent an elongation of the anterior skull (snout) and a wider posterior skull. Otherwise, the PACA gave results similar to the traditional PCA (above). For example, an additional 21% of the variation was summarized by the second PC axis, where positive loadings reflect skulls that are shorter in length and narrower in width. Less than 3% of trait variation was explained by each of the remaining axes.

Phy‐PCA: Results from a third PCA that created axes taking phylogenetic relationships into account (Revell 2009) differed from the other two PCAs by switching the two main axes. This PCA captured 71% of trait variation in the first PC axis, which reflected skulls with relatively longer anterior skulls (snout) and shorter heights. The second PC axis described 26% of the variation, with positive values reflecting overall longer and wider skulls. This axis carried little information about skull height. The remaining axes each explained < 7% of the trait variation.

We found relatively weak phylogenetic signal in overall skull shape (*K*_mult_ = 0.2, *p* < 0.01, effect size = 3.2), skull centroid size (*K*_mult_ = 0.3, *p* < 0.01, effect size = 1.9), relative skull length (Pagel's *λ* = 0.1, *p* = 0.07), skull width (Pagel's *λ* = 0.3, *p* < 0.01), and skull height (Pagel's *λ* = 0.2, *p* < 0.08), as would be expected if adaptive evolution has been driving phenotypes away from an expectation based on the phylogeny alone.

### 3.3 Modularity and Integration of the Skull

We found a high level of integration among skull components estimated for all our modular hypotheses including the tripartite model (CR = 0.88), the anoline model (CR = 0.93), and the mammalian model (CR = 0.94), suggesting that the elements of the male *Sceloporus* skull are evolving largely in concert. This was also confirmed by our phylogenetic integration test which yielded nearly identical results. The tripartite model showed an average pairwise partial least squares (PLS) correlation of 0.88 (*p* = 0.001) between the three modules, the anoline model had a PLS correlation of 0.94 (*p* = 0.001), and the mammalian model had a PLS correlation of 0.95 (*p* = 0.001). Nevertheless, our test of modularity found that the tripartite model (*Z*_CR_ = −5.85, *p* < 0.01) best fit our data compared to the anoline model (*Z*_CR_ = −3.36, *p* < 0.01), the mammalian model (*Z*_CR_ = −3.58, *p* < 0.01), and the null model (*Z*_CR_ = 0.00, *p* < 0.01), suggesting that parts of the skull are also evolving independently to some degree.

## 4 Discussion

Our results found little phylogenetic signal in these data and that body size was a good predictor of skull shape, with large‐bodied species having evolved more robust skulls (short snout lengths and wider posterior skulls) compared to smaller bodied species. In *Sceloporus* lizards, body size has increased over evolutionary time in several independent episodes (Rivera et al. 2021), and our results also suggest that skull evolution has slowed in larger bodied species as compared to smaller bodied species. Additionally, we found that the two PCAs that incorporate phylogeny in different ways yielded similar results to the non‐phylogenetic PCA. For the PACA, this implies that the morphological variation in the *Sceloporus* skull is driven by a high phylogenetic signal. This is likely driven by body size as body size was a good predictor of skull shape and body size had a high phylogenetic signal (Rivera et al. 2021). We show that most of the interspecific variation in the skulls of male *Sceloporus* lizards was concentrated in two parts: the length of the anterior part of the skull, namely the snout, possibly impacting sensory systems such as olfaction (Dawley 2017), and the width of the posterior part of the skull, like the posterior jaw, potentially influencing bite force (Herrel et al. 2001). Additionally, skull height also differed among species, albeit to a lesser degree, possibly contributing to bite force or habitat use. Finally, although we found limited evidence of the jaw, anterior, and posterior skull components evolving independently of each other (a tripartite model), skull structures have been evolving largely in concert rather than as independent modules, suggesting that allometry may be the primary driver of skull shape evolution rather than selection.

Although *Sceloporus* lizard skulls are like those of other vertebrates in many ways, they also differ in significant aspects. For example, *Sceloporus* lizard skulls are typical of most vertebrates in that interspecific variation emphasizes differences in snout length and is linked to body size (e.g., Cardini and Polly 2013; Felice, Pol, and Goswami 2021). This is unlike lacertid lizards, for example, in which the main axis of variation reflects the size of the orbits (Hipsley and Müller 2017). However, in most vertebrates, smaller bodied animals have shorter, rounder faces (e.g., Cardini and Polly 2013; Mitchell, Sherratt, and Weisbecker 2024; Stayton 2005), the opposite of what we found here. The link between body size and facial shape has been attributed to evolutionary allometry and this pattern is widespread across vertebrates, especially among domesticated mammals, and which may be explained by basic biomechanical properties during ontogeny (Cardini and Polly 2013; Emerson and Bramble 1993). In larger bodied animals, selection for skull shapes that can produce strong bite forces for foraging may be relaxed because larger jaws can bite as strongly as smaller jaws in an absolute sense due simply to allometry (Mitchell, Sherratt, and Weisbecker 2024). Here, we found instead that larger bodied *Sceloporus* species tend to have wider skulls with short snouts, whereas smaller bodied species have narrower skulls and elongated snouts. Our results are more congruent with those for amphibians in which larger bodied species have wider skulls with larger occipital regions and stronger jaws required for the consumption of larger prey (e.g., Carla Bardua et al. 2021; Isip, Jones, and Cooper 2022). Although *Sceloporus* lizards are diet generalists, elongated snouts or large jaws may reflect other behavioral demands and additional studies are needed to explore these possibilities.

### 4.1 Evolutionary Patterns of Skull Shape

Previous work has suggested that correlated traits may limit morphological evolution (Juha and Björklund 2004) while morphological modules allow for coordinated variation to arise allowing taxa to explore novel morphospace (Hendrikse, Parsons, and Hallgrímsson 2007). However, Sanger et al. (2012) suggest that these hypotheses may only hold true at an evolutionary time scale and different mechanisms may be at play at a geological time scale or in the short‐term. Like in some *Anolis* species (Sanger et al. 2012), we found a high degree of integration so that large species have shorter, more robust heads compared to smaller species (long, narrow heads), independent of evolutionary lineage. This pattern suggests that the integrated skull of *Sceloporus* is a result of selective pressures rather than phylogenetic constraints, evidenced by the convergence in skull shape among distantly related species. For example, large species may share similar selective demands (e.g., feeding ecology or fighting) on skull morphology leading to convergence on a robust skull shape. Likewise, the gracile skull shape of small species may be due to similar ecological demands, like using tight crevices to hide from predators. On the other hand, these patterns may also be explained by developmental changes where similar developmental processes were at play in different lineages leading to the pattern seen here, but this has yet to be studied in *Sceloporus*.

Importantly, we found that stochastic processes (Brownian motion) did not explain the evolution of linear measurements of the skull. Instead, body size best explained the patterns of skull length while we were not able to detect a statistical difference between a global regime and the body size regime models for skull width and height. For skull length, we found that larger species had a shorter skull length optimum while small species had a longer skull optimum. This may have consequences on behavior and feeding, discussed below. This pattern was mirrored by the evolutionary rates of the linear measurements. Species of different body sizes had varying rates of evolution for skull length while skull width and height had a single rate of evolution across the clade (Table 2). This may indicate that skull width and height may be more conserved or that selective pressures (i.e., stabilizing selection) to maintain the same relative width and height are strong. Conversely, skull length differed between species and body size implying that *Sceloporus* of different sizes are under different selective pressures for skull length. This mosaic of evolutionary patterns that generated skull shape diversity in *Sceloporus* is similar to that of early squamates undergoing a diversification event (Watanabe et al. 2019). In squamates, elevated rates of evolution were found at specific anatomical sites for specific lineages, like the frontal bone of dibamids, iguanians, and snakes. This may have been a consequence of niche vacancy after the Permian–Triassic extinction event. Here, we found that evolutionary rates have a mosaic pattern in *Sceloporus*, which has also undergone a rapid diversification event ~20 MYA (Leaché et al. 2016). It seems like periods of diversification may contribute to differences in evolutionary rates across the skull in squamates, perhaps facilitating the exploration of new niches and reinforcing the diversification event.

### 4.2 Skull Shape and Ecology

We found that small‐bodied species possessed elongated and narrow skulls while large‐bodied species had shorter and wider skulls. These differences in head shape may be driven by bite force. Herrel et al. (2001) noted that head morphology and bite force are directly related so that as SVL, head width, and head height increase, so does bite force. This is because larger head dimensions indicate larger bones, which increase the surface area for muscle attachment. The increased area where muscles can attach leads to larger jaw muscles that can amplify bite force production (De Meyer et al. 2019). Bite force, in turn, directly influences what prey lizards can consume. Lizards of species with wider and shorter heads should be able to consume a wider array of prey, including larger prey, compared to those with narrower and longer heads, potentially allowing species with more robust heads to explore a wider breadth of niche space. For example, robust skull species like *S. magister* and *S. jarrovii* can eat coleopterans, lepidopterans, hymenopterans, and smaller lizards (Galindo‐Gil et al. 2015) or have a greater reliance on hard prey like coleopterans, as is the case with *S. horridus* (Serrano‐Cardozo, Lemos‐Espinal Julio, and Smith 2008). Furthermore, there may added selective pressures to increase bite force in robust‐headed species that depend on hard prey because some insect taxa show a positive allometric relationship in the elytra thickness (Asgari et al. 2021). Similar patterns can be found in *Gambelia sila* , which has been shown to prey on orthopterans, coleopterans, hymenopterans, dipterans, hemipterans, and other lizards (Germano, Smith, and Tabor 2009). On the other hand, lizard species with gracile heads (long and narrow), like *S. occidentalis* and *S. jalapae*, consume a less diverse array of prey, including lepidopterans and orthopterans (Galindo‐Gil et al. 2015) or soft‐bodied prey, like termites, as their primary food resource (Serrano‐Cardozo, Lemos‐Espinal Julio, and Smith 2008). The gracile shape of the skull seems to limit the breadth of prey a lizard can consume, which may limit other important aspects of a lizard's biology like where it can live and which species it can be in sympatry with.

The reason why a robust skull, namely shorter rostrums, leads to strong bite forces is because the jaw is a third‐class lever and by shortening the rostrum, the fulcrum is moved closer to the load requiring less effort to produce a force. Therefore, when a large species with a short rostrum bite, it can produce an even larger force than what is merely gained by having a larger skull. Moreover, bite force must increase with increasing size (called the Allomeric Bite Coefficient) as is seen in larger mammals (Mitchell, Sherratt, and Weisbecker 2024). This same shortened snout shape found in large *Sceloporus* species can also be found in the highly specialized lizard species, *Amblyrhyncus cristatus*, which uses its blunt shape to increase the surface area contact with rocks while scrapping off algae (Paparella and Caldwell 2022).

Head shape may also be driven by intraspecific competition as bite force can be important for fending off male competitors in lizards (Herrel, Meyers, and Vanhooydonck 2002; Lailvaux et al. 2004; VanHooydonck et al. 2005). Males with relatively larger heads outcompete males with relatively smaller heads because large‐headed males produce stronger bites (Herrel, McBrayer, and Larson 2007; Lailvaux et al. 2004; VanHooydonck et al. 2005). We found that large‐bodied males tend to possess shorter, wider, and sometimes taller heads, which may produce a stronger bite than that of males from smaller bodied species, even when corrected for size (Herrel et al. 2001). This may be due to larger species being under stronger sexual selection compared to smaller species, but this has yet to be explicitly tested in *Sceloporus* lizards. The selective pressure imposed on male head shape via male–male interactions may also have other consequences. Because selection via competition increases head size in males but not necessarily females (Herrel et al. 2001), this could lead to sexual dimorphism in head shape and size (Roig‐Fernandez 1997), which remains to be tested. In fact, one of the limitations of this study is the lack of female skull information which may help identify which selective pressures are shared between the sexes and which selective pressures are unique to each sex. Moreover, using information on both males and females, we can begin to tease apart the nuanced shape differences between the sexes that may be impossible to detect using traditional linear morphometrics. Lastly, including females can also shed light on different allometric patterns between the sexes, which is lacking in the literature.

In addition to resources and mate acquisition, head shape may be driven by habitat use. *Sceloporus* can be found in a wide variety of habitats and environments (Hews and Martins 2013) that impose different selective pressures on morphology. For example, Galindo‐Gil et al. (2015), found that lizards of more arboreal *Sceloporus* species have more shallow, flattened heads compared to those from terrestrial or rock‐dwelling species. Flattening of the skull and body may be an advantage in a vertical habitat because it helps individuals to better balance and allows lizards to use crevices to avoid predation (Lappin, Hamilton, and Sullivan 2006; Vitt et al. 1997). The flattening of the skull in response to arboreality may limit other phenotypic aspects, like bite performance, which can be compensated by evolving a wider head or shorter rostrum. Here, we found that the evolution of skull height was best explained by a single global optimum, which may be the reason why skull width and length differ among lineages.

### 4.3 Skull Shape and Communicative Signaling

In addition to wide diets and habitat use, *Sceloporus* lizards have a broad repertoire of behavior, including communicative signaling, that ranges from visual head bobs and push‐ups to more chemosensory behavior like tongue flicks and depositing of femoral pores (Hews and Martins 2013). Moreover, *Sceloporus* also uses visual cues, like their hallmark ventral patch, to convey information (Ossip‐Drahos et al. 2018; Romero‐Diaz et al. 2019). The ventral patch is often used during male territorial disputes as a signal of aggression (Cooper and Burns 1987; Martins 1993; Martins, Ord, and Davenport 2005; Ossip‐Drahos et al. 2018). However, the ventral patch has been independently lost several times across the *Sceloporus* lineage, perhaps because the patch is too conspicuous to predators against particular physical backgrounds (Hews and Martins 2013; Ossip‐Drahos et al. 2016; Wiens 2001). Most species that have lost the ventral patch are small in body size and exhibit compensatory increases in chemical signals (Romero‐Diaz et al. 2021). The skull elongation in small species found here may facilitate chemical perception as chemical perception can be increased by larger snouts or by thickening the olfactory and vomeronasal epithelia (Dawley 2017; but see Erudaitius et al. 2023). Thus, skull shape and its links to body size may be tightly linked to behavior and communicative signaling.

## 5 Conclusions

Despite *Sceloporus* being a well‐studied clade, we have revealed novel information about their evolution using μCT scans, traditional linear morphometrics, geometric morphometrics, and a comparative approach. Our study encompasses about half the known species diversity of the lineage allowing for in‐depth investigation of the clade. We have shown that allometry plays an important role in the skull shape evolution of *Sceloporus* lizards. However, once allometric effects were accounted for, there was evidence that selective forces also contribute to the skull shape evolution in *Sceloporus* lizards and that these differences may have profound consequences for their diet, ecology, intraspecific interactions, and behavior. What yet remains to be understood is whether the patterns seen here apply to the rest of the anatomy in *Sceloporus*. Specifically, whether *Sceloporus* morphologies are a mosaic of complex forces that are working simultaneously and sequentially through evolutionary time or whether they are a consequence of simple allometric scaling in this generalist lizard group.

## Author Contributions

**Julio A. Rivera:** conceptualization (equal), data curation (equal), formal analysis (equal), writing – original draft (equal). **Jesualdo A. Fuentes‐G.:** conceptualization (equal), data curation (equal), methodology (equal), writing – original draft (equal). **Emília P. Martins:** conceptualization (equal), funding acquisition (equal), supervision (equal), writing – original draft (equal).

## Conflicts of Interest

The authors declare no conflicts of interest.

## Supporting information


Table S1

Table S2

## Data Availability

The 3‐dimensional images of the skulls will be freely available on MorphoSounce (https://www.morphosource.org/) and part of the openVertebrate Thematic Collection Network. The R code used for the analyses will be made available on JAR's GitHub page (https://github.com/juliorivera85).
