# Supplementary material for: Evolutionary Links Between Skull Shape and Body Size Suggest Allometric Forces and Selection at Work in a Generalist Group of Lizards
Source: Ecol Evol. 2024 Nov 17;14(11):e70594. doi: 10.1002/ece3.70594 (PMC11569864; doi:10.1002/ece3.70594)
Supplement: Supplementary file 1 — Table S1 [file ECE3-14-e70594-s001.docx]

Table S1. Metrics for each *Sceloporus* species used in this study, including (1) body size measures including Snout-Vent length (SVL) and size category (L=large, M=medium, and S=small), (2) linear head measurements (Head Length, Width, and Height), (3) museum and catalogue number from which each specimen was borrowed, and (4) ventral patch category. Museums are: UTA = Amphibian and Reptile Diversity Research Center at the University of Texas at Arlington, UMVZ = The Museum of Vertebrate Zoology at the University of California Berkeley, CUMNH = the Museum of Natural History at the University of Colorado Boulder, MSB = Museum of Southwestern Biology at The University of New Mexico, and UWBM = Burke Museum at the University of Washington.

| Species | SVL (mm) | Size | Head length (mm) | Head width (mm) | Head height (mm) | Collections | Cat no. | Ventral  Coloration |
| --- | --- | --- | --- | --- | --- | --- | --- | --- |
| *S. acanthinus* | 85.44 | L | 14.9 | 12.0 | 7.0 | UTA | R-41784 | colorful |
| *S. adleri* | 58.93 | M | 15.1 | 11.1 | 7.0 | UTA | 11523 | colorful |
| *S. aeneus* | 49.83 | S | 11.6 | 8.3 | 5.5 | UMVZ | 79910 | colorful |
| *S. angustus* | 86.73 | L | 21.1 | 12.7 | 8.5 | UMVZ | 96763 | colorful |
| *S. arenicolus* | 58.30 | S | 13.7 | 9.1 | 6.5 | UMVZ | 180320 | colorful |
| *S. bicanthalis* | 39.88 | S | 10.5 | 7.3 | 4.6 | UMVZ | 106413 | white |
| *S. carinatus* | 52.88 | S | 13.8 | 8.7 | 6.1 | UTA | R-41453 | white |
| *S. chrysostictus* | 53.31 | S | 13.1 | 9.2 | 6.1 | CUMNH | 28914 | colorful |
| *S. clarkii* | 100.89 | L | 22.5 | 18.7 | 11.5 | UMVZ | 244261 | colorful |
| *S. consobrinus* | 57.72 | M | 14.8 | 10.8 | 6.7 | MSB | 75641 | colorful |
| *S. couchii* | 56.85 | M | 12.1 | 8.7 | 5.6 | UMVZ | 36762 | colorful |
| *S. cowlesi* | 62.58 | M | 15.1 | 11.2 | 7.1 | UWBM | 7381 | colorful |
| *S. cozumelae* | 55.25 | M | 14.1 | 8.1 | 6.1 | CUMNH | 16627 | white |
| *S. cyanogenys* | 125.15 | L | 29.8 | 21.6 | 12.6 | UTA | R-41213 | colorful |
| *S. dugesii* | 67.26 | M | 15.7 | 11.8 | 6.9 | UMVZ | 72188 | colorful |
| *S. edwardtaylori* | 63.29 | L | 16.8 | 14.0 | 8.5 | CUMNH | 45607 | white |
| *S. formosus* | 69.98 | M | 18.0 | 13.5 | 8.8 | UTA | R-51751 | colorful |
| *S. graciosus* | 55.26 | S | 12.6 | 9.5 | 6.2 | UMVZ | 30125 | colorful |
| *S. grammicus* | 69.32 | M | 16.1 | 11.5 | 7.8 | UMVZ | 78269 | colorful |
| *S. grandaevus* | 53.46 | S | 14.4 | 8.9 | 5.7 | UMVZ | 117339 | colorful |
| *S. hartwegi* | 64.05 | M | 15.9 | 12.2 | 7.6 | UTA | R-51534 | colorful |
| *S. horridus* | 96.42 | L | 23.2 | 18.5 | 12.5 | UMVZ | 78257 | colorful |
| *S. hunsakeri* | 65.64 | M | 16.2 | 12.9 | 8.4 | UMVZ | 104226 | colorful |
| *S. internasalis* | 80.07 | L | 19.3 | 14.8 | 9.5 | UTA | R-41861 | colorful |
| *S. jalapae* | 42.08 | S | 10.6 | 7.0 | 4.4 | UTA | R-51780 | colorful |
| *S. jarrovii* | 83.35 | M | 22.4 | 16.3 | 9.2 | UMVZ | 59085 | colorful |
| *S. licki* | 76.85 | L | 17.2 | 12.3 | 8.4 | UMVZ | 236292 | colorful |
| *S. magister* | 101.07 | L | 23.6 | 19.0 | 12.1 | UMVZ | 235867 | colorful |
| *S. malachiticus* | 84.73 | L | 20.4 | 16.5 | 9.9 | UMVZ | 40167 | colorful |
| *S. melanorhinus* | 87.48 | L | 21.8 | 16.2 | 10.7 | UMVZ | 160081 | colorful |
| *S. merriami* | 51.83 | S | 15.9 | 11.6 | 7.6 | UTA | R-16723 | colorful |
| *S. minor* | 77.84 | M | 19.4 | 15.0 | 9.1 | UTA | 11740 | colorful |
| *S. mucronatus* | 78.70 | L | 19.7 | 14.1 | 8.8 | UTA | R-51782 | colorful |
| *S. nelsoni* | 54.20 | M | 14.0 | 9.9 | 6.4 | UMVZ | 50700 | colorful |
| *S. occidentalis* | 80.64 | M | 19.1 | 12.3 | 8.5 | UMVZ | 61245 | colorful |
| *S. orcutti* | 96.99 | L | 21.5 | 16.8 | 11.6 | UMVZ | 13577 | colorful |
| *S. parvus* | 39.67 | S | 9.6 | 6.7 | 4.1 | UMVZ | 129326 | colorful |
| *S. poinsetti* | 94.14 | L | 22.9 | 18.7 | 10.6 | UMVZ | 229646 | colorful |
| *S. prezygus* | 93.29 | L | 22.5 | 19.1 | 10.4 | UTA | R-41433 | colorful |
| *S. pyrocephalus* | 68.32 | M | 15.6 | 10.8 | 7.5 | UTA | R-59892 | colorful |
| *S. scalaris* | 58.63 | S | 13.5 | 9.5 | 6.8 | CUMNH | 49665 | colorful |
| *S. scitulus* | 70.26 | M | 17.7 | 13.6 | 8.5 | UTA | R-51622 | colorful |
| *S. siniferus* | 49.93 | S | 12.9 | 7.9 | 5.5 | UMVZ | 78293 | white |
| *S. slevini* | 47.47 | S | 11.8 | 8.3 | 5.1 | UMVZ | 96983 | colorful |
| *S. smaragdinus* | 79.35 | M | 16.7 | 12.5 | 7.7 | UMVZ | 159452 | colorful |
| *S. spinosus* | 99.16 | L | 23.5 | 19.4 | 12.6 | UTA | 11800 | colorful |
| *S. squamosus* | 51.60 | S | 13.5 | 9.2 | 5.9 | UMVZ | 40260 | white |
| *S. taeniocnemis* | 77.15 | L | 19.4 | 14.6 | 9.0 | UTA | R-27388 | colorful |
| *S. teapensis* | 59.46 | M | 12.4 | 8.1 | 5.3 | UTA | 8932 | colorful |
| *S. torquatus* | 98.90 | L | 22.6 | 18.5 | 11.5 | UMVZ | 68956 | colorful |
| *S. tristichus* | 71.46 | M | 16.5 | 12.7 | 8.0 | UWBM | 7499 | colorful |
| *S. undulatus* | 60.79 | S | 14.7 | 11.0 | 7.3 | CUMNH | 24643 | colorful |
| *S. utiformis* | 52.48 | S | 12.7 | 8.3 | 5.7 | UMVZ | 236299 | colorful |
| *S. variabilis* | 52.81 | M | 13.0 | 9.5 | 6.3 | UTA | R-53181 | colorful |
| *S. virgatus* | 50.54 | S | 12.8 | 9.5 | 6.3 | UMVZ | 225525 | white |
| *S. woodi* | 51.53 | S | 12.6 | 8.9 | 5.8 | UWBM | 7273 | colorful |
| *S. zosteromus* | 103.73 | L | 26.1 | 20.0 | 13.4 | UMVZ | 117390 | colorful |
