# Supplementary material for: Evolutionary Links Between Skull Shape and Body Size Suggest Allometric Forces and Selection at Work in a Generalist Group of Lizards
Source: Ecol Evol. 2024 Nov 17;14(11):e70594. doi: 10.1002/ece3.70594 (PMC11569864; doi:10.1002/ece3.70594)
Supplement: Supplementary file 2 — Table S2 [file ECE3-14-e70594-s002.docx]

Table S2. Description of the anatomical position of landmarks placed on each *Sceloporus* skull, and designation in modularity hypotheses. These landmarks were also used to create the morphospace for the traditional PCA, the Phy-PCA, and the PACA. We placed landmarks on the dexter (right) side first then the sinister (left) side. Each landmark was assigned to either A = the posterior part of the skull, B = the anterior part of the skull, or C = the mandible to test for modularity. We also derived linear measures from these landmarks so that skull length was the distance between landmarks 15 and 37, skull width was the distance between landmarks 50 and 51, and skull height was the distance between landmarks 12 and 43.

| Landmark Number | Anatomical description of landmark position | Tripartite | Anole | Mammal |  |
| --- | --- | --- | --- | --- | --- |
| 1 | Most superior and anterior external point along the alveolar margin of Premaxilla (looks like Foramen) | A | A | A |  |
| 2 | Most superior and anterior external point along the alveolar margin of Premaxilla (looks like Foramen) | A | A | A |  |
| 3 | Most posterior point of the lateral enlargement (meets with maxilla) of Prefrontal bone | A | A | A |  |
| 4 | Most posterior point of the lateral enlargement (meets with maxilla) of Prefrontal bone | A | A | A |  |
| 5 | Most anterior point of nasal-maxilla suture seam (external) of Nasal bone | A | A | A |  |
| 6 | Most anterior point of nasal-maxilla suture seam (external) of Nasal bone | A | A | A |  |
| 7 | Inferior extremity of anterior orbital process of Preorbital bone | A | A | A |  |
| 8 | Inferior extremity of anterior orbital process of Preorbital bone | A | A | A |  |
| 9 | Most posterior point of prefrontal-frontal suture seam (along the orbital margin) of Frontal bone | A | A | A |  |
| 10 | Most posterior point of prefrontal-frontal suture seam (along the orbital margin) of Frontal bone | A | A | A |  |
| 11 | Most posterolateral point, near the junction of the frontal-postfrontal-parietal suture seam of Frontal bone | A | B | B |  |
| 12 | Most posterolateral point, near the junction of the frontal-postfrontal-parietal suture seam of Frontal bone | A | B | B |  |
| 13 | Most posterior external point of Squamosal bone | A | B | B |  |
| 14 | Most posterior external point of Squamosal bone | B | B | B |  |
| 15 | Most posteroventral (often broadest) point of the "ventral peg" of Squamosal | B | B | B |  |
| 16 | Most posteroventral (often broadest) point of the "ventral peg" of Squamosal | B | B | B |  |
| 17 | Most ventral point of the side of the basal tubercle of Basioccipital | B | B | B |  |
| 18 | Most ventral point of the side of the basal tubercle of Basioccipital | B | B | B |  |
| 19 | Most posteroventral point (closest to quadrate) of Pterygoid | B | B | B |  |
| 20 | Most posteroventral point (closest to quadrate) of Pterygoid | B | B | B |  |
| 21 | Most anterior point of right Basipterygoid process | B | B | B |  |
| 22 | Most anterior point of right Basipterygoid process | B | B | B |  |
| 23 | Most anterior point where Epipterygoid structure meets Palatine bone | B | B | B |  |
| 24 | Most anterior point where Epipterygoid structure meets Palatine bone | B | B | B |  |
| 25 | Most superior point of Epipterygoid structure | B | B | B |  |
| 26 | Most superior point of Epipterygoid structure | B | B | B |  |
| 27 | Interior foramen on Zygomatic arch | B | B | B |  |
| 28 | Interior foramen on Zygomatic arch | B | B | B |  |
| 29 | Interior foramen of Jugal bone | B | B | B |  |
| 30 | Interior foramen of Jugal bone | B | B | B |  |
| 31 | Posterior most point where the Pterygoid and Maxilla meet | B | B | A |  |
| 32 | Posterior most point where the Pterygoid and Maxilla meet | B | B | A |  |
| 33 | Most anterior point where posterior-most tooth meets Maxila | B | B | A |  |
| 34 | Most anterior point where posterior-most tooth meets Maxila | B | B | A |  |
| 35 | Most inferior external point along the alveolar margin. | A | A | A |  |
| 36 | Most inferior external point along the alveolar margin. | A | A | A |  |
| 37 | Most anterior point of the lower jaw | A | A | A |  |
| 38 | Most posterior point of the Dentary and Surangular suture | C | B | A |  |
| 39 | Most posterior point of the Dentary and Surangular suture | C | B | A |  |
| 40 | Most anterior point of the Dentary and Surangular suture | C | B | A |  |
| 41 | Most anterior point of the Dentary and Surangular suture | C | B | A |  |
| 42 | Most posterior point of the Dentary and Angular suture | C | B | A |  |
| 43 | Most posterior point of the Dentary and Angular suture | C | B | A |  |
| 44 | Most superior point of the Coronoid process of the dentary | C | B | B |  |
| 45 | Most superior point of the Coronoid process of the dentary | C | B | B |  |
| 46 | Inferior point of Coronoid process of the dentary ridge | C | B | A |  |
| 47 | Inferior point of Coronoid process of the dentary ridge | C | B | A |  |
| 48 | Foramen that is just posterior of the Coronoid process of the dentary | C | B | A |  |
| 49 | Foramen that is just posterior of the Coronoid process of the dentary | C | B | A |  |
| 50 | Point where the Quadrate and Dentary meet | C | B | A |  |
| 51 | Point where the Quadrate and Dentary meet | C | B | A |  |
| 52 | Posterior and exterior most point of the outer process of the Articular | C | B | B |  |
| 53 | Posterior and exterior most point of the outer process of the Articular | C | B | B |  |
| 54 | Posterior and interior most point of the outer process of the Articular | C | B | B |  |
| 55 | Posterior and interior most point of the outer process of the Articular | C | B | B |  |
| 56 | Posterior and interior most point of the inner process of the Articular | C | B | B |  |
| 57 | Posterior and interior most point of the inner process of the Articular | C | B | B |  |
| 58 | Point where the Articular and Quadrate meet | C | B | B |  |
| 59 | Point where the Articular and Quadrate meet | C | B | B |  |
| 60 | Inferior most point of the interior Coronoid process of the dentary ridge | C | B | A |  |
| 61 | Inferior most point of the interior Coronoid process of the dentary ridge | C | B | A |  |
| 62 | Anterior point of the foramen in the interior jaw | C | B | A |  |
| 63 | Anterior point of the foramen in the interior jaw | C | B | A |  |
| 64 | Anterior point of the foramen that is posterior to the Coronoid process | C | B | A |  |
| 65 | Anterior point of the foramen that is posterior to the Coronoid process | C | B | A |  |
| 66 | Posterior point of the anterior jaw foramen near the tip of the snout | C | A | A |  |
| 67 | Posterior point of the anterior jaw foramen near the tip of the snout | C | A | A |  |
| 68 | Posterior point after the most posterior tooth | C | B | B |  |
| 69 | Posterior point after the most posterior tooth | C | B | B |  |
